# Research on the death psychology among Chinese during and after the COVID-19 pandemic

**DOI:** 10.1038/s41598-024-53673-1

**Published:** 2024-02-06

**Authors:** Xiaowen Li, Yuanqing He

**Affiliations:** 1 College of Education, Sehan University, Yeongam, 58425 Korea; 2 College of Educational Sciences, Anhui Normal University, Wuhu, 241000 China

**Keywords:** Psychology, Human behaviour

## Abstract

Under the threat of the novel coronavirus, people are compelled to contemplate some ultimate existential questions, such as life and death. This study collected texts related to the death psychology from Sina Weibo, and after data cleaning, a total of 3868 Weibo texts were included. Study 1 employed grounded theory from qualitative research to explore the core categories and evolutionary mechanisms of people's psychology when facing death threats in the context of the pandemic. Study 2 utilized big data mining techniques such as topic mining and semantic network analysis to validate the effectiveness of the death psychology theory developed in qualitative research. The findings demonstrate that within the “Emotion–Cognition–Behavior-Value” framework, the implications of death threats manifest in four aspects: death anxiety, death cognition, coping efficacy, and sense of meaning. As time progresses, the study of death psychology can be segmented into four distinct phases: the tranquil phase prior to lifting pandemic restrictions, the threat phase at lifting pandemic restrictions onset, the coping phase mid-lifting pandemic restrictions, and the reformative phase post-lifting pandemic restrictions. The calculated outcomes of topic mining and semantic network analysis corroborate the coding results and theories derived from the grounded theory. This reaffirms that data mining technology can be a potent tool for validating grounded theory.

## Introduction

The COVID-19 Pandemic has led to a significant number of fatalities and widespread panic globally. Its high contagion and the current lack of effective treatments have instilled a fear of death among individuals, posing a serious threat to their physical and mental health. Recent data from a study involving 5000 international participants suggest a direct correlation between an individual's perception of COVID-19 and increased levels of anxiety, along with deteriorating mental health conditions^1^. The concept of death psychology encompasses a complex psychological response that includes emotion, cognition, and behavioral intention. This response arises when individuals confront death or related events^2^. Nevertheless, it remains inconclusive as to whether the threat of death precipitates fear, anxiety, personal growth, or instigates a re-evaluation of life's meaning^3^. In the realm of traditional Confucian thought, the notion of death intertwines closely with ancestor worship and filial piety. Consequently, the Chinese populace’s perception of death extends beyond mere fear, evolving into a more nuanced sensation of awe. Since the founding of the People's Republic of China, campaigns launched by the Party and the state to modify funeral customs, alongside the influence of Western pragmatist culture, have prompted the public to confront death more pragmatically and with increased diversity. However, the disintegration of traditional ideologies and the absence of firm new concepts have resulted in traits such as collective avoidance, the propensity for sudden emotional outbursts, and vulnerability of anxiety buffering mechanisms when Chinese individuals face death^4^.

Since the onset of the pandemic three years ago, the Chinese government has steadfastly implemented a strategy known as “dynamic zero,” an approach centered on rigorous epidemic prevention and control. This strategy has involved extensive nucleic acid testing, enabling the tracking and containment of virtually all infected individuals and their close contacts. However, the institution of a new policy on December 7, 2022, marked the termination of the “dynamic zero” strategy, effectively lifting epidemic restrictions. This policy shift was followed by a substantial increase in the epidemic, characterized by a sharp rise in the daily number of infections and death toll. Amid this environment of heightened mortality and escalating panic, a clear understanding of people's psychological response to the threat of death is crucial. Therefore, delineating the foundational parameters of death psychology research can facilitate more nuanced future theoretical inquiries. Furthermore, it can contribute to the development of tailored, effective social psychological services.

**Figure 1:**
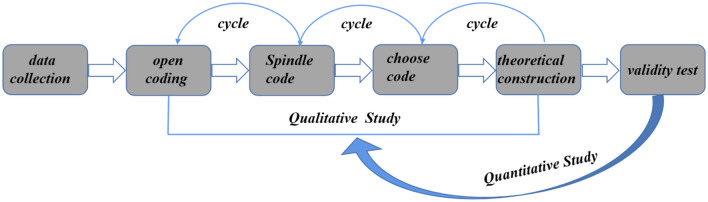
Research flow chart.

Previous research on the death psychology often employed questionnaire methods, but questionnaires have limitations in terms of quantity and are subject to the subjective interference of the respondents, especially for sensitive topics like death psychology, where there is a greater likelihood of participant bias towards social expectations^5–7^. Therefore, the data for this study were sourced from Weibo posts. Weibo posts are the spontaneous and natural expressions of opinions by Weibo users in their daily lives, continuously and non-invasively recorded on Weibo. Such posts are considered more objective and thus more likely to reduce social expectation bias^8^. Considering the exploratory nature of the study, Study 1 employed grounded theory from qualitative research to explore the core categories and evolutionary mechanisms of people’s psychology when facing death threats under the backdrop of the pandemic. To overcome the limitation of personal subjective bias in grounded theory, Study 2 utilized big data mining techniques such as topic mining and semantic network analysis to validate the effectiveness of the death psychology theory formed in qualitative research (as shown in Fig. 1).

## Data collection

Amid the epidemic, social media has emerged as a primary conduit for the public to access information, voice opinions, and share emotions^9^. It has proven effective in gauging public sentiment and implementing epidemic-related interventions. According to the Blue Book of China's Social Mentality, approximately 74.3% of posts on Weibo—a prominent social media platform—are expressive of personal emotions. Notably, negative emotions propagate more readily within the Weibo network than positive ones. During the epidemic, Weibo initiated several epidemic-related topics such as “What to do if you're anxious staying at home,” “What to do if you always suspect that you're sick,” and “What would make you feel anxious?” These emotionally-charged topics spread swiftly across the Internet. Contrasting with conventional research data collection methods such as questionnaire surveys and in-depth interviews, studies by Heikinheimo et al. demonstrate that social media data alleviates the problem of reporting time lag caused by physical limitations. Moreover, given the dynamism of social media platforms and the presence of personal information tags, more effective data can be obtained through the monitoring of temporal and spatial patterns^10,11^.

Consequently, this research established a research database by crawling text data from Sina Weibo. Using Weibo's advanced search function, texts from December 1, 2022, to March 1, 2023, were initially collected using the keywords “death” and “epidemic,” resulting in 10,273 text data. Upon manual inspection, we found that a significant portion of the collected information consisted of irrelevant content such as dialogues, lyrics, and advertisements. To clean the dataset, we employed the Harbin Institute of Technology stop-word list for text filtering, ultimately obtaining 3868 valid data entries for inclusion in the study.

## Study 1: theoretical construction based on grounded theory

### Methods

Text data analysis is fundamentally a “bottom-up” process. Its primary objective is to inductively ascertain the rules of association and transformation of behaviors from the actions detailed in the original text. It further seeks to abstract higher-level principles to explicate the progression from individual behaviors to specific outcomes, as well as to elucidate how various social interaction modes coalesce to create distinctive, recognizable social processes^12^. As per Strauss et al., text analysis in qualitative research entails the dissection of raw materials through three stages of encoding: open coding, spindle coding, and choose coding^13^. Open coding, or first-level coding, every word, sentence, and paragraph of the imported raw data is analyzed using NVIVO software. With “death psychology” as the core concept, sentence-by-sentence coding refines the keywords in the raw data, culminating in the first level of conceptualization. Spindle coding, or second-level coding, forms categories, properties, and dimensions based on open coding, and constructs and tests the relational rules between categories. The objective here is to develop rules from first-level coding and to cluster or segregate first-level coding according to these rules, thereby accomplishing the second level of conceptualization. Choose coding, or third-level coding, further generalizes and categorizes axial coding, forming explanatory theories. The process tests whether the concepts discerned in spindle coding have reached theoretical saturation. Concepts that have achieved theoretical saturation are deemed the core categories of third-level coding. Upon completion of third-level coding, the analysis process revisits first- and second-level coding for iterative verification. Through continuous verification of the entire text data, from open coding through to choose coding, theoretical saturation of node integration and classification is finally achieved, establishing the final theoretical construct. The intrinsic logic of these three stages embodies the scientific induction process of “induction–iterative verification–re-induction–iterative verification”.

### Results

Through comparative analysis of the materials and three-level coding using NVIVO software, we interpret death psychology in the context of the COVID-19 pandemic from four dimensions: “Emotion-Cognition-Behavior-Value”. Consequently, we construct four core categories: Death Panic, Death Cognition, Coping Efficacy, and Sense of Meaning (Table 1), as well as the evolutionary process of death psychology.

**Table 1:** Four categories of death psychology.

Core category	Choose coding	Spindle code	Open coding	Reference point
Death panic	Emotional dysregulation	Avoid	Unable to face, emotional isolation	103
		Emotional response	Death anxiety, death scare, Emotional overflow, emotional contagion, asphyxia	451
	Emotional stability	Actively face	Put aside, ignore, emotional desensitization	187
Death cognition	Cognitive dysregulation	Unexpected death	Indeterminate death, accidental death, death of the elderly, unceremonious death, innocent death, conformant death, death in develop life	225
		Out of control	Coronary pneumonia symptoms, basic illness	87
		Broken relationship	Separation of loved ones, companion separation, die alone, interpersonal conflict, implicate relatives and friends, separation empathy	112
	Cognitive stability	Intimacy buffer	Parent–child communication, husband and wife communication	141
		Human interaction buffer	Ritual gathering, peer support, interpersonal avoidance	115
		Compensatory gratification for the relationship	Intimacy deepens, strangers deepen	377
		Relationship enhancement	Intimacy, peer support, help others, common experience	211
Coping efficacy	Coping efficacy dysregulation	Uncertain information	Prevention is uncertain, cure uncertain, uncertain death figures	236
		Excessive behavior	Excessive hoarding, unreasonable collection of resources	196
	Coping efficacy stability	Information confirmation	Access to authoritative information, rule obedience, rational expectations	108
		Materials determined	Hoarding, government funding	116
		The course of treatment is determined	Preventive control, successful medical treatment, symptom control, posthumous planning	197
		Runaway prevention	Death avoidance, cherish the existing, fulfill last wish	97
Sense of meaning	Traumatic stress response	State of nothingness	Unrealistic, conformity, give up survival, believe in fate, pay attention to the body, nothingness, numbness, vulnerability, value shock	246
		Values	Educational experience, cultural background, Can't survive	115
	Post-traumatic growth	Individual mentality	Optimism, doubtful, rational understanding, experience understanding	294
		Meaning reconstruction	Scientific knowledge learning, tragic comparison, Positive meaning, sense of responsibility, religious belief, belief in survival	254

Over time, death psychology evolves through four stages: the Tranquil phase prior to lifting pandemic restrictions, the Threat phase at lifting pandemic restrictions onset, the Coping phase mid-lifting pandemic restrictions, and the Reformative phase post-lifting pandemic restrictions.

#### Tranquil phase

Prior to the lifting of restrictions, individuals maintained a dynamic equilibrium in the face of death, characterizing a period of calm. Confronted with the death of someone distantly related, they often exhibited a nonchalant attitude: “Just ignore it for now. It really won't provoke a substantial emotional response in me”. Confronted with the potential threat of their own mortality, individuals typically respond passively. For instance, some study subjects reported having “written wills”. Professional roles also influence responses to death; medical groups, when facing mortality, often choose to “engage in discussion and summarization of the specific (death) case”, thereby maintaining a sense of control and stability.

#### Threat phase

With the lifting of pandemic restrictions and the subsequent explosive increase in infection numbers and epidemic-related fatalities, the impact of death threat can be summarized across four aspects: Emotional Dysregulation, Cognitive Dysregulation, Coping Efficacy Dysregulation, and Traumatic Stress Response.

#### Emotional dysregulation

Nearly every participant alluded to the death panic instigated by the epidemic's outbreak, using phrases like “so scary” and “unable to face it”. Numerous cases reported elevated levels of emotional panic, often accompanied by symptoms such as “inability to sleep at night”, and in more extreme cases, “fear to go out” and “hesitation to meet people”.

#### Cognitive dysregulation

This chiefly presents as unanticipated death, physical loss of control, and fractured relationships. Unlike the predictable mortalities of the elderly or late-stage cancer patients, the death threats posed by COVID-19 are challenging to foresee and accept. Patients may “not want to die, but they might be informed in two weeks that they could die, causing immense distress to both patients and their families”. Individuals with underlying diseases worry about losing physical control upon infection, such as experiencing sensations of suffocation and pain. For instance, a participant's father who “had weak lungs” was perceived to be “quite dangerous”. The impact of death threats on the social level manifests as anxiety about broken relationships. On the one hand, death leads to “long-term separation”. Concurrently, under the influence of the COVID-19 epidemic, even the death of strangers can trigger vicarious grief. One participant reflected on seeing a deceased person's wife “crying heartbreakingly behind the car, at that moment, I felt on the verge of tears”.

#### Coping efficacy dysregulation

This primarily exhibits as uncertainty of information and excessive behaviors. A common sentiment is the perceived mystery surrounding the virus, with doubts such as whether “its transmission route is contact or aerosol”. Respondents also acknowledge the uncertainty, stating “it's there, and it's very dangerous.” In China, the previously implemented dynamic zero policy often led to city lockdowns, instigating a public tendency to stockpile supplies. However, under the threat of death, many study participants displayed excessive hoarding and unreasonable accumulation of resources. Statements such as “my fridge can't fit anything else,” and “there's stuff everywhere at home” exemplify this behavior.

#### Traumatic stress response

The threat of death during the COVID-19 pandemic can trigger an existential crisis in respondents, primarily manifesting as a diminishing of self-psychological representation, culminating in a psychological “void state” and a physiological “survival state”. The specter of death compels individuals to ponder existential matters profoundly. Death is often characterized as “a kind of vast void, you don’t know what it is.” After witnessing numerous deaths, individuals perceive that “life is truly fragile.” Moreover, when coping mechanisms falter, people’s internal subjectivity diminishes, leading them to “leave it to fate, to destiny.” Externally, they place heightened emphasis on physical health, with the sentiment that “physical health is more important than anything else.”

Additionally, for some COVID-19 patients and frontline workers, the thinking and meaning-making functions at the psychological level have been supplanted, resulting in a biological “survival state,” centered on “preserving their own lives” and “saving the lives of others.” Individuals “won't contemplate the future at all; survival becomes the primary focus.” For those with close family members infected with COVID-19, their immediate concern pivots to helping relatives secure a hospital bed “as soon as possible.”

#### Coping phase

After a phase of emotional turmoil, public panic regarding the novel coronavirus has subsided, and the overall level of comprehension and responsiveness has progressively improved.

#### Emotional stability

Individuals are confronting the situation proactively rather than passively accepting it. A greater number of people are adopting a mindset of “let it be” and “going with the flow.”

#### Cognitive stability

Cognitive coping strategies can be enacted in two ways: relationship buffering and relationship enhancement. Firstly, the relational disruption incited by the threat of death can be mitigated by “spending more time with children” and “increasing family time.” Frontline workers can gain social support by “sharing experiences” and “encouraging one another” within their professional circles. Communication between individuals with shared experiences can also alleviate feelings of fear, as “everyone is facing a common enemy,” and “we are comrades-in-arms.” Moreover, for medical professionals who need to maintain rationality, when dealing with patients, they adopt the strategy of “treating him solely as a patient” to insulate themselves from potential emotional fluctuations.

#### Coping efficacy stability

This can be developed in four primary ways: information certainty, material certainty, treatment process certainty, and prevention of loss of control. Firstly, obtaining authoritative information, such as “professional studies by the health commission,” allows individuals to feel a sense of control. Doctors “organize learning” and processes gradually become “standardized”. “Preventing the virus is like a computer program, if you do 'a', it inevitably leads to 'b', and 'b' inevitably leads to 'c'.” Secondly, the availability of sufficient resources also provides the public with a sense of control. In the early stages of the outbreak, some interviewees “rushed out to buy rice and oil, and a lot of vegetables. Then they just sat at home comfortably.” The government's large-scale and timely supply of resources also alleviated the sense of losing control, “the country gathered such a large force to contribute, the state did not want a penny, the disease was cured, and lives were saved.” Additionally, doctors who had already experienced many death events at work before the outbreak have become desensitized, “so we are used to it.” If treatment is ineffective, the individual's planning for what comes after death can also alleviate the loss of control brought about by the threat of death, “if we go, I tell him (relatives) how to educate the children,” and “how to face the occurrence of such things.” Finally, an individual's planned execution of their last wishes can also prevent the loss of control. “Hope to fulfill all the promises before dying.” By indirectly avoiding the issue and immersing oneself in “enjoying the present moment,” one can also gain a sense of control.

#### Post-traumatic growth

Coping at the level of meaning can be developed through two primary aspects: meaning reconstruction and individual mentality. (1) Meaning Reconstruction: By continuously expanding professional scientific knowledge, individuals can renew their explanatory system and strategies for dealing with the threat of death. For example, medical staff “discuss and summarize cases.” Some members of the public gain a kind of psychological comfort by comparing their plight with others. For instance, “So many people online are saying whose elderly people have died. The elderly in my family are still alive, maybe I am luckier.” Positive attributions to death events can also bring relief, stating: “On the one hand, it may be a disaster, but on the other hand, it is indeed a good change for the people of Wuhan.” (2) Individual Mentality: For frontline workers, strengthening their sense of responsibility can alleviate the meaning crisis brought about by the threat of death, “Wearing this uniform, so I should do this.” Individuals with religious beliefs deal with the threat by reinforcing their faith, “If you believe in God, He will take you to heaven.” For the general public, a strong will to live is an effective strategy when dealing with the threat of death, “As in Chairman Mao's era, there was often a saying called 'Man conquers nature,' which is the power of the spiritual atomic bomb.” Individuals who have been cured show an “unselfish side” by performing acts of selfless aid, such as “donating” supplies or “donating blood plasma,” and so forth.

#### Reformative phase

After the coping period, individuals gradually enter a new period of tranquility, during which the psychological mechanisms for facing death are reshaped. Individuals no longer merely evade or passively accept the impact of death. Instead, they “cherish life more,” “pay more attention to their bodies and strengthen exercise,” and proactively focus on life itself to gain a new sense of life's meaning. The approach to interpersonal relationships also becomes more proactive, with “interactions friendlier than before.” A new understanding and sense of meaning towards the world is also rebuilt, “I originally thought the world was gray, and suddenly the world is bright again.” However, for survivors who have lost loved ones, “surviving feels even more painful.”

## Study 2: validity testing based on data mining

### Methods

Established methods for validity testing in qualitative research encompass raw data inspection and expert evaluation^13,14^. Raw data inspection entails reviewing the concepts and relationships derived from the analysis. Here, “concepts” originate from the raw data, while “relationships” emanate from the model developed through layered induction from the raw data. Expert evaluation, on the other hand, assesses the validity of research outcomes via expert review, hence providing an additional layer of validity testing.

One of the key strengths of grounded theory is its inductive nature—it does not begin with the testing of pre-existing hypotheses but rather fosters the use of data and its subsequent analysis to formulate concepts and theories. However, this approach has a potential pitfall—it is susceptible to “personal subjective bias”, whereby investigators’ preconceptions may unduly influence their findings. Traditional methods of validity assessment have not adequately addressed this phenomenon. Thus, this study advocates for the application of big data mining techniques as a data-driven means to verify the validity of the resultant theory.

The topic coherence score is a metric that evaluates the semantic consistency of high-probability words associated with each topic generated by a model. A higher topic coherence score suggests superior model performance^15^. Given its efficacy in assessing model quality, this study utilizes the topic coherence score to determine the optimal number of topics. If the coherence score elevates concurrently with an increasing number of topics, and eventually plateaus at a certain value, the number of topics corresponding to the highest coherence score prior to stabilization is considered the optimal topic count.

Semantic network analysis serves to extract high-frequency co-occurring words at different stages following the removal of epidemic restrictions. This method constructs a co-occurrence matrix of high-frequency words, thereby revealing the focal points of public death psychology during the research period^16^. High-frequency words are further divided into clustering subgroups based on the results of the co-occurrence matrix. The evolving mechanism of public death psychology at distinct stages is manifested through changes in the number of high-frequency word nodes and the interconnections between subgroup nodes.

### Results

#### Topic modeling

**Figure 2:**
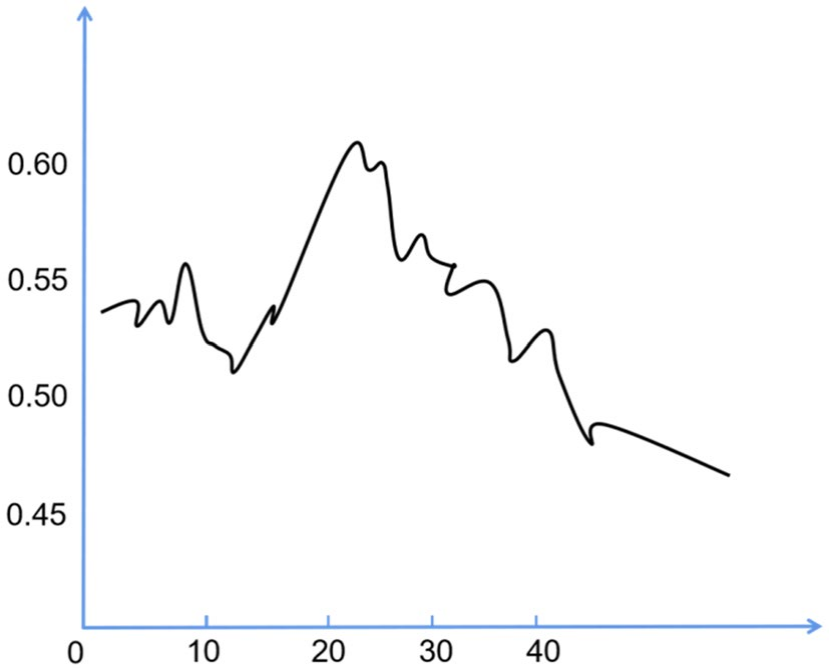
Topic consistency score chart.

This article uses Latent Dirichlet Allocation (LDA) for topic mining by the pyLDAvis library in Python. This article determines the number of topics using topic coherence scores. As shown in Fig. 2, if the coherence score increases with the increase of the number of topics until it stabilizes at a certain value, then the number of topics corresponding to the highest coherence score before stabilization is the optimal number of topics.

As can be seen from Fig. 2, the topic coherence score gradually increases until it reaches its maximum when the number of topics is 20. Subsequently, as the number of topics increases, the topic coherence score gradually stabilizes amidst fluctuations. Therefore, according to the topic coherence score, the text content in this article is ultimately generated into 20 topics, as shown in Table 2.

**Table 2:** Topic model description table.

Number	Topic keywords	Topic description	Topic strength
1	Unable to face; can’t sleep; crazy; mood; unban; dare; afraid to go out	Avoid	0.11623506
2	Scared; headache; anxiety; depression; lockdown; annoying; hurts; over; asphyxia; How to do; what should i do; do what; epidemic; virus	Emotional response	0.11365468
3	Palliation; uncomfortable; ignore; hold; unblock	Actively face	0.13141391
4	Indeterminate death; accidental death; coronary pneumonia symptoms; family separation; conformant death; separate; death; unknown; uncertainty	Unexpected death	0.09806952
5	Basic illness; uncomfortable; spreading	Out of control	0.23748256
6	Separation; miss him; unable to meet; date; bye; elder; unblock	Broken relationship	0.05923408
7	Close; great	Intimacy buffer	0.09290911
8	Daily life; home	Human interaction buffer	0.16924376
9	Gathering; nice guy	Compensatory gratification for the relationship	0.16014126
10	Friends; common experience	Relationship enhancement	0.06580284
11	Uncertain; gossip; afraid; number of deaths; fake	Uncertain information	0.18781868
12	Excessive behavior; hoarding; full	Excessive behavior	0.15280253
13	Listen; official notice; Zhong Nanshan	Information confirmation	0.09688606
14	Control; sufficient supplies; got used to	Materials determined	0.11365468
15	Ibuprofen; one week	The course of treatment is determined	0.37511391
16	Vacation; how much longer; last wish	Runaway prevention	0.12236952
17	Fragile; nothingness; fate; numbness	State of nothingness	0.13368256
18	Unanimous; help; save	Values	0.21909408
19	Fate; ignorance is bliss; happiness comes first; insights into life; figured out	Individual mentality	0.43590911
20	Do well; future; selfless	Meaning reconstruction	0.36324376

#### Topic evolution in semantic network analysis

**Figure 3:**
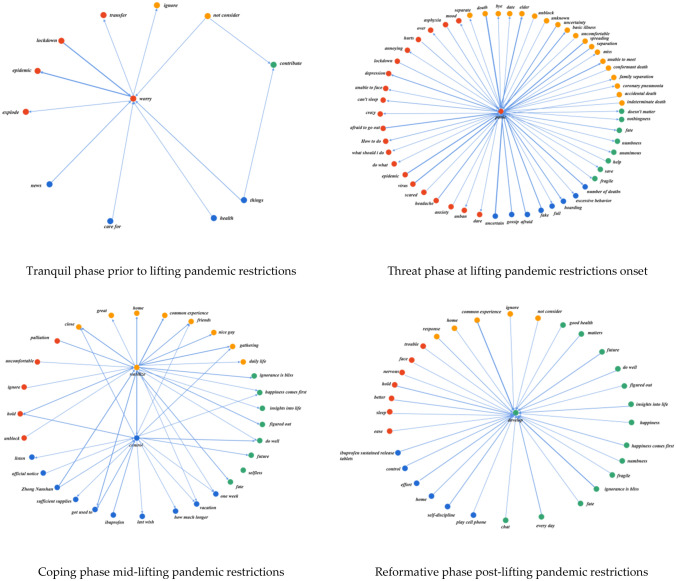
Topic evolution at various stages.

The Network X package in Python is used to extract high-frequency co-occurring words from the four stages. To more intuitively display the popular topics of public discussion at each stage, a high-frequency word co-occurrence matrix is further constructed and visualized, with the results seen in Fig. 3. According to specific interpretations, they can be divided into four clustered subgroups, with nodes of the same color belonging to the same category. The four subgroups are: emotion category (red), cognition category (yellow), behavior category (blue), and value category (green). The lines between the nodes represent the connections between the high-frequency words.

The structural composition of the semantic clustering network transitions across four distinct stages, as illuminated by shifts in the inter-nodal relationships. Preceding the lifting of constraints, societal focus on the psychological repercussions of pandemic-associated fatalities remained relatively subdued, fostering a climate of comparative tranquility. As restrictions began to ease, the interconnections among nodes within each subgroup tightened. The emotion category encompassed the most nodes, with 'panic' serving as the central node, indicating a pervasive public dread arising from the rampant escalation of the pandemic-induced mortality rate. As the easing phase progressed, the mid-stage was characterized by the emergence of dual-core nodes in cognition and behavior categories, termed ‘stabilize’ and ‘control’ respectively. This shift signifies that, post the initial panic phase, the public began acquiring an evolved perspective of the pandemic, fostering the development of more robust response strategies. In the later phases of constraint relaxation, public comprehension of the pandemic, coupled with the efficacy of their response strategies, displayed further stabilization and progression. Concurrently, the reinforcement of herd immunity was observed as widespread infections prevailed. Transitioning into the post-pandemic era, psychological mechanisms employed by individuals for dealing with mortality entered a phase of reconstruction. This was notably manifested in the value category as ‘develop’, signifying a societal recalibration of attitudes towards death in the wake of the epidemic’s cessation.

### Ethics statement

The study protocol was approved by the Ethics Committee of Anhui Normal University.

## Discussion

In the face of uncertainty, humans endeavor to augment their sense of control to mitigate perceived threats. The self-expansion theory, viewed from an individual’s standpoint, posits that individuals enhance their sense of self-efficacy by integrating others into their self-construct, facilitating more effective threat management^17^. Meanwhile, the uncertainty-identity theory, from a group perspective, contends that uncertainty can induce a state of immobilization and distress while simultaneously triggering a drive to alleviate this uncertainty. This motivates individuals to employ strategies such as group identification to diminish the ambiguity^18,19^. Moreover, it has been observed that uncertain information disseminated through media can engender a sense of control loss among the public. The rapid and continual influx of epidemic-related data can lead to misinformation, as the availability of information may be misinterpreted as its validity. In situations where information is ambiguous or cognitive resources are constrained, unverified rumors can fill cognitive gaps, potentially facilitating rumor proliferation. Thus, the procurement of authoritative information can psychologically instill individuals with a sense of control. Death anxiety, on a separate note, is frequently associated with the fear of losing physical control and power^20,21^. A meta-analysis of 1087 articles concerning hypochondriasis revealed a positive correlation between death anxiety and hypochondriasis^22^.

The results of this study align with prior research, indicating that death threats can intensify intimate relationships and incite anxiety regarding potential relationship dissolution. The Terror Management Theory posits that mortality reminders, also known as mortality salience, spur investment in intimate relationships as a means of alleviating death anxiety^23^. Such intimate relationships serve as the primary buffer against this anxiety^24^. Research focusing on phobias has uncovered a positive correlation between death anxiety and separation anxiety, signifying that an increase in death anxiety concurrently escalates anxiety regarding the prospect of separation from significant others^25^. Furthermore, a negative correlation has been observed between death anxiety and the quality of parental emotional relationships and secure attachment styles, suggesting that lower levels of death anxiety correspond with enhanced quality of these relationships^26^.

Chinese culture, characterized by vertical collectivism, emphasizes the individual's identity as a component of a larger group. The common Chinese philosophical acceptance of soul immortality, intertwined with the unbreachable divide between yin and yang after death, suggests that a family member's demise is perceived as not only an individual extinction, but also as a potential threat to the entire family's continuity. This is often counteracted by the perpetuation of lineage. Research conducted among Hong Kong residents revealed that compared to Western counterparts, they display heightened concern regarding the responses of loved ones and the prospect of separation from significant individuals when contemplating health-related matters^27^. This evidence underscores the significance, both symbolically and tangibly, of emphasizing Chinese cultural norms in preventing relationship breakdowns.

Death, the ultimate inevitability for humans, is an unalterable truth. Faced with the threat of death, individuals are compelled to discard the illusion of immortality and reflect on the essence of life and death. Evolutionary psychology perceives death as a fundamentally neutral event^28^. Nonetheless, individual interpretations of death can evoke emotional responses, consequently influencing a person's approach to life and preparations for death^29^. Absence of a framework to understand death may precipitate a value conflict when death is confronted.

From the standpoint of time management, research indicates that reminders of mortality prompt individuals to shift focus towards future-oriented thinking, fostering positive adaptive responses in time management^30^. Research concerning the consideration of future consequences (CFC) reveals that individuals with high CFC scores, due to greater consideration of future outcomes, are likely to foster healthier lifestyles encompassing balanced nutrition, physical exercise, and safety practices^31^. Thus, an appropriate understanding of death can instigate beneficial lifestyle modifications. Previous research identified a correlation between high rumination and diminished mental health levels, as well as negative adaptation post-bereavement^32^. Some researchers suggest that individuals of high compassion, especially those who have experienced trauma, are more proficient at discerning and empathizing with the suffering of others, more inclined to offer assistance, and thus more prone to engage in acts of rescue, donation, blood donation, and volunteering. These behaviors are crucial for self-assistance, mutual aid, and post-disaster reconstruction^33^.

## Conclusion

This research delves into the transformation of mortality-related psychology against the backdrop of COVID-19 restriction easing, utilizing grounded theory as the methodological framework. Based on the temporal progression and death-associated factors such as emotions, cognition, behavior, and values, four stages can be delineated: the tranquility phase prior to restriction lifting, the mortality threat phase in the early stage of relaxation, the coping phase during the middle stage, and the restructuring phase in the later stage. The psychological implications of death triggered by the pandemic onset can be bifurcated into two trajectories from the vantage points of mental, social, and physical realities: loss of control and stability. The 'loss of control' trajectory encompasses emotional volatility, cognitive instability, diminished coping efficacy, and traumatic stress responses. In contrast, the 'stability' trajectory comprises emotional equanimity, cognitive steadiness, stable coping efficacy, and post-traumatic growth. Subsequent to the threat and coping stages, individuals gradually transition into a renewed period of serenity, during which the psychological mechanisms employed for grappling with death undergo reshaping.

This research dismisses the conventional validation technique associated with grounded theory, critiquing its excessive dependence on the researcher's subjective interpretation, which may foster “researcher bias”. In lieu of this, the study employs LDA topic modeling and semantic network analysis to authenticate the preliminary theory's credibility. Findings suggest that the quantitative outcomes derived from topic mining and semantic network analysis robustly corroborate the coding outcomes and theoretical constructs of grounded theory. This implies the potential of data mining technologies as efficacious validation tools for grounded theory.

The limitations and future directions of this study are as follows: The research population is exclusively Weibo users from Mainland China, making it challenging to extend the results to other cultural groups. In the future, there is a need to enhance the focus on cross-cultural studies in the psychology of death. Finally, this study employs an exploratory grounded theory approach, which may not delve deeply enough into the psychological mechanisms of death. Subsequent research should combine experiments and other methodologies to further investigate the internal mechanisms of death psychology.

## Data Availability

The datasets used during the current study are available from the first author on reasonable request.
